# AI-powered drone-based automated inspection of FAST

**DOI:** 10.1038/s41377-023-01094-6

**Published:** 2023-03-03

**Authors:** Lijun Wang

**Affiliations:** Dalian University of Technology, Dalian, Liaoning, China,

**Keywords:** Imaging and sensing, Astronomical optics

## Abstract

Automated optical inspection of FAST is realized by exploiting advances in drone technology and deep-learning techniques. The AI-powered drone-based automated inspection is time-efficient and reliable, which guarantees the stable operation of FAST.

The Five-hundred-meter Aperture Spherical radio Telescope (FAST), also known as the “China Sky Eye”, is the world’s largest single-dish radio telescope^1^. Its optical geometry is outlined in Fig. 1a. The reflector is a partial sphere of radius *R* = 300 m. The planar partial spherical cap of the reflector has a diameter of 519.6 m, 1.7 times larger than that of the former largest radio telescope. The large reflecting surface makes FAST the world’s most sensitive radio telescope. It was used by astronomers to observe, for the first time, fast radio bursts in the Milky Way and to identify more than 500 new pulsars, four times the total number of pulsars identified by other telescopes worldwide. More interesting and exotic objects may yet be discovered using FAST.

**Fig. 1:**
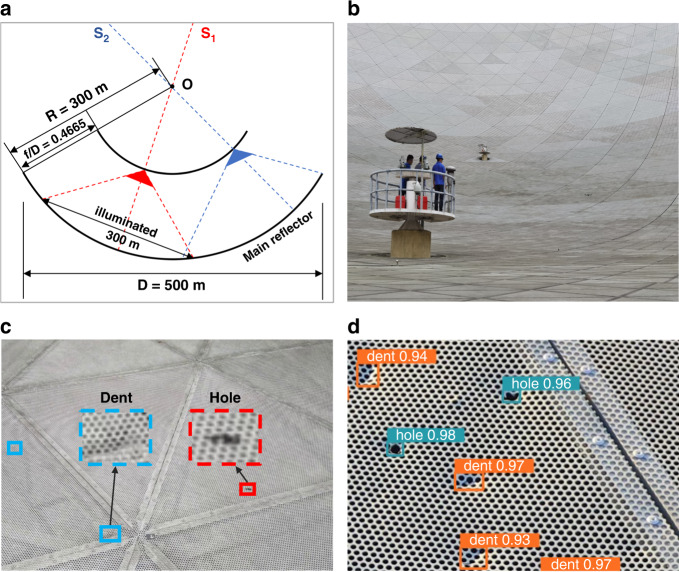
Visualization results. **a** FAST’s optical geometry. **b** Automated optical inspection at FAST. **c** Illustration of surface defects (dent and hole). **d** Results of defect detection

However, each coin has two sides. A larger reflecting surface is more prone to external damage due to environmental factors. The FAST reflector comprises a total of 4450 spliced trilateral panels, made of aluminum with uniform perforations to reduce weight and wind impact. Falling objects (e.g., during extreme events such as rockfalls, severe windstorms, and hailstorms) may cause severe dents and holes in the panels. Such defects adversely impact the study of small-wavelength radio waves, which demands a perfect dish surface. Any irregularity in the parabola scatters these small waves away from the focus, causing information loss.

The rapid detection of surface defects for timely repair is hence critical for maintaining the normal operation of FAST. This is traditionally done by direct visual inspection. Skilled inspectors climb up the reflector and visually examine the entire surface, searching for and replacing any panels showing dents and holes. However, this procedure has several limitations. Firstly, there is danger involved in accessing hard-to-reach places high above the ground. Secondly, it is labor- and time-consuming to scrutinize all the thousands of panels. Thirdly, the procedure relies heavily on the inspectors’ expertize and is prone to human-based errors and inconsistencies.

The remedy to the shortcomings of manual inspection at FAST is automated inspection. A recent publication in *Light: Advanced Manufacturing* by Li et al.^2^ made the first step towards automating the inspection of FAST by integrating deep-learning techniques with drone technology. As a first step, the research team integrated deep-learning techniques with the use of drones to automatically detect defects on the reflector surface. Specifically, they began by manually controlling a drone equipped with a high-resolution RGB camera to fly over the surface along a predetermined route (Fig. 1b). During the flight, the camera captured and recorded videos of the surface condition. One benefit of the advanced flight stability of drones is that the recorded videos can capture much information on surface details. Moreover, thanks to the GPS device and the RTK module onboard the drone platform, every video frame can be tagged with the corresponding drone location with centimeter-level accuracy. The physical locations of the panels that appear in each frame can thus be determined.

Previous works involving defect detection using aerial imagery were primarily designed to detect large defects and were not reliable for detecting very small defects^3–8^. In contrast, this work aims to inspect the large surface of FAST from on high. The surface defects in drone imagery exhibit large-scale variation and high inter-class similarity (Fig. 1c). To tackle the above challenges, the research team introduced a simple yet effective cross-fusion operation for deep detectors, which aggregates multi-level features in a point-wise selective manner to help detect defects of various scales and types (Fig. 1d). The cross-fusion method is lightweight and computationally efficient, particularly valuable features for onboard drone applications. Future work will implement the algorithm on embedded hardware platforms to process captured videos onboard the drone, and to make the inspection system more autonomous and robust.
